# Impact of statistical poly(propylene co-ethylene) on the thermo-mechanical properties of high-density polyethylene

**DOI:** 10.1038/s41598-024-77467-7

**Published:** 2024-11-08

**Authors:** Mohamed H. Darweesh, Bernhard Stoll, Safaa H. El-Taweel

**Affiliations:** 1 Engineering and Materials Science Department, German University in Cairo, New Cairo City, Egypt; 2 Abteilung Angewandte Physik, University of Ulm, Ulm, Germany; 3 Chemistry Department, Faculty of Science, Cairo University, Orman- Giza, 12613 Egypt

**Keywords:** High-density polyethylene, Statistical poly(propylene-co-ethylene), Rheological behavior, DSC, Stress-strain test, Polarized optical microscopy, Polymer chemistry, Polymer characterization

## Abstract

A series of high-density polyethylene and a statistical copolymer of poly(propylene-co-ethylene) blends in a wide range, namely (0, 10, 20, 30, 40, 50, 60,70, 80, 90, 100) abbreviated as HDPE/VM were systematically investigated by using a rheometer, differential scanning calorimetry (DSC) measurements, polarized optical microscopy (POM) and tensile tests. Rheometer results show that adding VM decreases dynamic viscosity, storage, and loss modulus. Han plot shows that HDPE and VM are compatible and miscible in the range from 20 to 60 VM % in the molten state. DSC results show little nucleation effect of VM on HDPE (HDPE’s melt crystallization temperature shifts 2 °C higher). Moreover, a linear composition dependence of ∆cp ∆Hc, ∆Hm shows that PE and VM are most probably compatible in the molten state in composition range from 20 to 60%. However, upon crystallization, the VM and PE domains occur distinctively. The results of the tensile tests demonstrated a decrease in elastic modulus, yield stress, and ultimate tensile strength as VM content increased. At low VM content (less than 20%), high elongation at break was detected for the blends, and very fine spherulites of HDPE were found across the sample by POM.

## Introduction

High-density polyethylene (HDPE) is a prevalent thermoplastic polymer characterized by its lightweight, cost-effectiveness, non-toxicity, excellent humidity resistance, and favorable mechanical properties^1^. HDPE is frequently utilized in rotational molding, extrusion of pipes, and the production of blown containers^3,12^. Large-sized polyethylene products are exposed to more complex loads and significant impacts during transportation and use^2–5^. Therefore, polyethylene needs to have superior fracture resistance and low-temperature toughness^6^. Two strategies for improving the toughness of HDPE have been mentioned: chemical modification and physical modification. The chemical modification involves grafting and crosslinking processes ^4,5^, while the physical modification involves blending with elastomers and adding fillers ^6,7^. It has been reported that HDPE-based materials with excellent performance can be efficiently and successfully produced by blending with natural rubber powder (NRP)^4^, ethylene-octene copolymer (POE)^6–8^, scrap rubber powder (SRP)^9,10^, ethylene-propylene-diene terpolymer (EPDM)^11,12^, and ethylene vinyl acetate copolymer (EVA)^13,14^. Takidis et al.^15^ reported that blends of low-density polyethylene (LDPE) and poly(ethylene-co-vinyl acetate) containing 18 wt% vinyl acetate units (EVA-18), were prepared by melt mixing using a single-screw extruder at 180 °C, were compatible across the entire range of compositions studied (25, 50, and 75 wt%). Faker et al.^16^ reported a decrease in the melting temperature of LDPE upon the addition of EVA, which is attributed to the partial miscibility between LDPE and EVA. Peon et al.^17^investigated the viscoelastic properties of PE/EVA blends. They analyzed the phase structure of the blends using linear viscoelastic data and predicted co-continuity at a composition of 60 wt% PE in the PE/EVA blend. Chen et al.al^14^. investigated the thermal and rheological properties of binary HDPE/ and EVA-18 blends. Their findings revealed that the interfacial interaction was stronger in HDPE-rich blends than in EVA-rich blends, indicating good compatibility. Additionally, they reported that the dilution effect of EVA-18 chains within the HDPE phase reduced the melting temperature of HDPE. Chen et al.^14^. reported that HDPE and EVA are likely miscible in the molten state, which can prevent molecular segregation during self-nucleation and annealing, forming stable co-crystals. Stelescu et al.^11^ investigated HDPE and EPDM blends’ thermal and mechanical properties. They discovered that HDPE’s crystal structure remained unchanged in the blends. However, when a compatibilizing agent was added to the blends, they observed a rise in both hardness and elongation at break. It is known that copolymers of PE and PP can act as compatibilizers.

Recently, ExxonMobil Chemical Co. developed a propylene-ethylene statistical copolymer, known commercially as Vistamaxx (VM), using advanced metallocene catalysis^18^. Due to its excellent mechanical properties and processability, Vistamaxx has increasingly gained attention as a toughening modifier for olefin polymers^19–21^. Li et al.^22^investigated the morphology, rheological, thermal, and mechanical properties of HDPE/VM blends with 90/10, 80/20, and 70/30 compositions. The SEM results revealed a phase-separated morphology, indicating that HDPE and VM are immiscible. They found that the presence of VM resulted in a slight reduction in the crystallinity of HDPE^22^.

In our previous manuscript^21^, PP/ VM blends with (100/0, 90/10,80/20, 70/30, 60/40, 50/50, 40/60,30/70,20/80,10/90,0/100) compositions were studied. It was found that VM is miscible with PP, and as the VM content was increased, the blend’s crystallization, elastic modulus, and tensile strength decreased, and the blends’ toughness increased. Therefore, this study explores the compatibility, crystallization behavior, and mechanical properties of HDPE blends with VM in a wide range, namely, 0, 10, 20, 30, 40, 50,60, 70, 80, 90, and 100%.

## Experimental part

### Materials

The high-density polyethylene Lupolen 6011 L, abbreviated as HDPE, from BASF (Germany), with a weight average molar mass Mw = 63 kg mo1^− 1^, Mw/Mn = 2 was used in this research. Its melt flow index (MFI) was 5.5 g/10 min (230 °C/2.16 kg).

ExxonMobil, USA, supplies the Vistamaxx TM 6202, which has an MFI of 20 g/10 min (230 °C/2.16 kg). This statistical copolymer, consisting of 85% propylene and 15% ethylene, has a molecular weight of Mn = 170 000. Here, it is shortened to VM.

The HDPE/VM blends for compositions containing 0, 10, 20, 30, 40, 50, 60,70, 80, 90, and 100% have been made. The series was made using a pineapple distributive mixer (Noztek Pro Touch) in a lab setting with a 12:1 screw length-to-diameter ratio. The screw has two heating bands and a diameter of 14 mm. Its length is 170 mm, separated into feeding, compression, and metering zones, and it has a mixing head that is 85 mm long and has two heating bands. 1.75 mm has a nozzle attached to it. The rpm was 20, and the temperatures ranged from 220 to 240 °C. Many test measurements by DSC on samples from different locations of the extrudate ensured that the material was homogeneous.

### Rotational rheometer

The Malvern company’s Bohlin Gemini type was utilized as an instrument. Oscillating rotation was seen at frequencies ranging from 0.02 Hz to 40 Hz. The strain 0.003. The disc’s thickness was 2 mm, and the polymer sample had a diameter of 15 mm. Since minimal rotation angles were selected, the polymer material’s deformation was consistently within the linear viscoelastic range. A stream of hot air, with a maximum temperature of 230 °C, was employed to regulate the sample’s temperature. Each time, a reading at 230 °C is taken initially, followed by smaller temperatures in steps. Readings at various frequencies and temperatures were taken using the same sample to avoid thickness and diameter determination uncertainties.

### Differential scanning calorimetry

One effective method for tracking the temperature and heat flow associated with polymer transitions as a function of temperature and time is differential scanning calorimetry (DSC). Q100 (TA Instruments, USA) was used to investigate the thermal transitions of blends. Before DSC measurements, calibration with standard indium was performed^23^. Nitrogen served as the measurement’s atmosphere. 8–10 mg of sample was cut from the extruded blend, placed in the aluminum DSC pan and Lids, and then sealed. In the first run, the samples were heated from − 50 °C to 200 °C at a rate of 10 °C/min to remove thermal history and achieve good contact between the sample and the DSC pan; therefore, no data of the first heating run were evaluated or considered. Then, the DSC sample was cooled at 10 °C/min to -50 °C, and the maximum melt crystallization temperature (T_c_) and its enthalpy (∆H_C_) were evaluated from this step. The second heating run was conducted from − 50 °C to 200 °C at 10 °C /min from − 50 °C to 200 °C. Thermal Transitions such as glass transition temperature (T_g_), its corresponding change in heat capacity ∆Cp, maximum cold crystallization temperature (Tcc), its cold crystallization enthalpy DHcc, and maximum melting temperature (Tm), and its melting enthalpy (∆H_m_) were evaluated using TA Advantage DSC software from the second heating run.

### Polarized optical microscopy

An Imager A1 polarized light optical microscope (Zeiss, Germany) with a digital camera was utilized to investigate the crystallization morphology of blends. After being placed between two thin glass slides and heated to 200 °C on a hot plate for three minutes, each sample was rapidly cooled to the required crystallization temperature. Both HDPE and its blends were crystallized at 119 °C. Polarized optical micrographs were taken after annealing for 60 min at 119 °C. Three samples for each blend were examined.

### Tensile test

Hot pressing in a sheet mold under vacuum at 190 °C for five minutes has produced tensile samples, which are gradually cooled to 20 °C. A cutting die ISO 527-2-Type 5 A was used to cut the dumbbell-shaped specimens (width = 4 mm, thickness ~ 2 mm, length L0 ~ 25 mm). It should be mentioned that the dog-bone samples used in this work are cut after crystallizing while cooling to room temperature. A universal testing machine, Z100 (Zwick, Germany), assessed the tensile properties (yield strength and elongation at break) at a strain rate of 10 mm/min and 25 °C by ISO 527 standard. Three measurements were made for every blend.

## Results and discussion

### Rheological behavior of HDPE/ VM

**Fig. 1:**
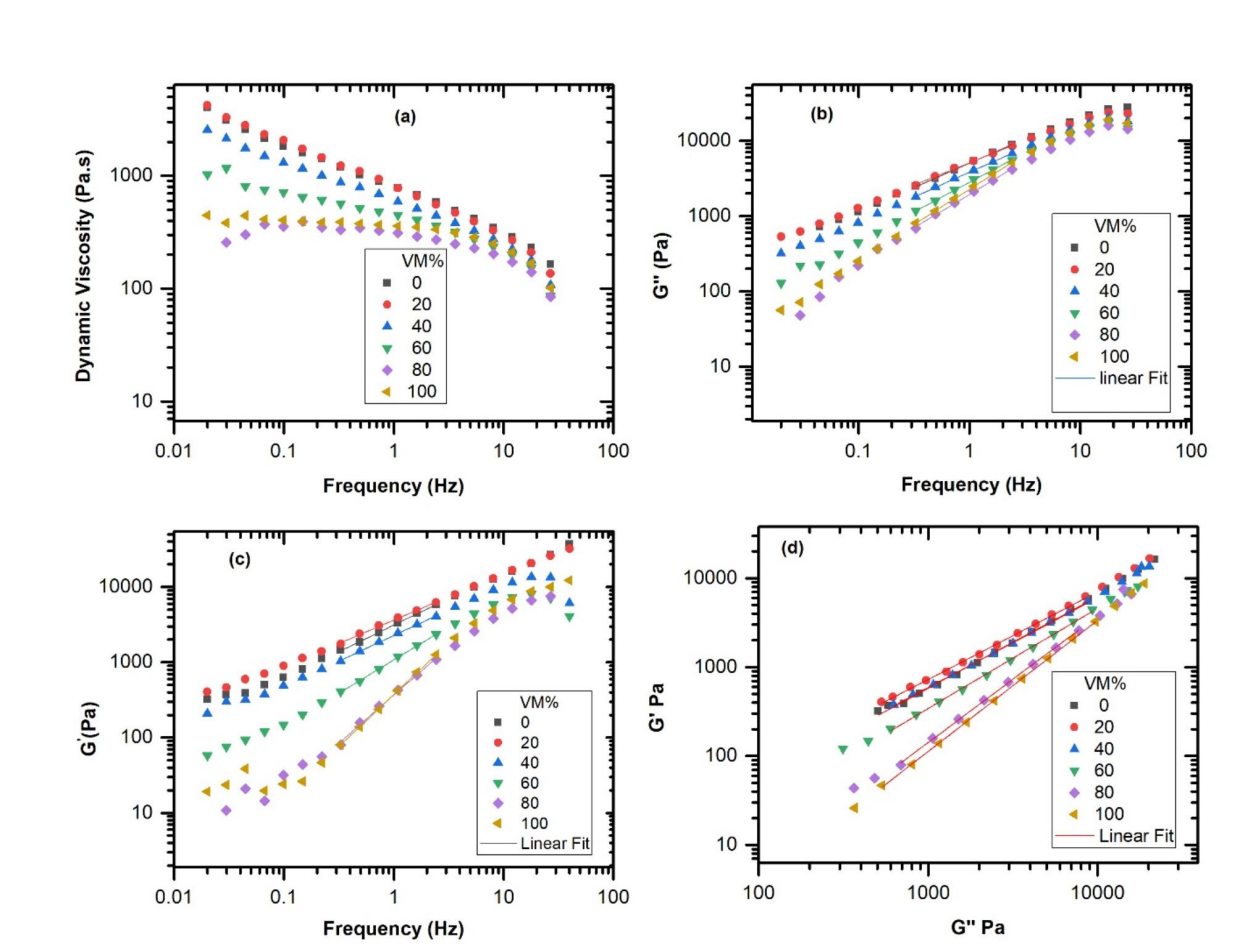
Frequency dependence of (**a**) complex viscosity, (**b**) loss modulus G″, (**c**) storage modulus, and (**d**) Han plot of pure HDPE, pure VM, and HDPE/VM blends.

The study of rheology involves examining how materials behave when subjected to external forces. Figure 1 represents the frequency dependence of complex viscosity, loss modulus G’, storage modulus of pure HDPE, pure VM, and HDPE/VM blends at 230 ^o^C. Dynamic viscosity versus frequency is a critical parameter in understanding the behavior of fluids under different conditions. At low frequencies, fluids with high dynamic viscosity will exhibit more resistance to flow, while at high frequencies, the resistance may decrease. This relationship is essential in various industries that can optimize processes and design more efficient systems. Dynamic viscosity of pure HDPE and pure VM versus frequency decreased with increasing frequency for both materials, as shown in Fig. 1a. However, the decrease rate was more pronounced for HDPE than for Vistamaxx. HDPE and its blends with VM showed shear thinning, while pure VM showed a Newtonian melt behavior with a Newtonian plateau. This difference in behavior may be attributed to the two materials’ molecular structure and chain flexibility. Adding VM to HDPE resulted in a gradual decline in dynamic viscosity values. This aligns with the fact that the HDPE had a lower MFI than the VM, as mentioned in the experimental part.

The storage and loss modulus of HDPE and HDPE/VM blends as a function frequency at 230°C are presented in Figs 1b, c. It was found that the loss modulus of the HDPE/VM blends with VM content higher than 20% was lower than that of pure HDPE in the frequency range. However, for HDPE/Vistamaxx (80:20), the loss modulus was slightly higher than that of pure HDPE. Additionally, the storage modulus of the blends exhibited a similar trend. The loss modulus is approximately proportional to the viscosity of the material being tested. This means that materials with higher viscosity will exhibit a higher loss modulus, indicating a greater tendency to dissipate energy as heat. At 230 ^o^C in the terminal range, the loss modulus G’’is higher than the storage modulus G’. The slope of log G’’ versus log frequency should be + 1 for a narrow molecular weight distribution. This has been demonstrated, for instance, by Macosko for SBR and polybutadiene^24^. For pure VM, it is slightly less than 1 (see Table 1), which indicates a relatively narrow distribution of molecular weights. For HDPE, the slope is about 0.6, which means a wide distribution of molecular weights. For instance, such slope was also observed in gels at very low crosslinking density, as mentioned by Macosk^24^, or in blends of two polystyrene fractions with different molecular weights, as observed by Pechhold et al.^25^.

**Table 1:** Slopes of G′ and G″ versus frequency and slope of Han plots for HDPE/VM blends.

Vm%	Slope of G′	Slope of G″	Slope han plot
0	0.7	0.64	1.08
20	0.62	0.59	1.05
40	0.68	0.66	1.01
60	0.88	0.78	1.06
80	1.27	0.88	1.198
100	1.38	0.95	1.156

On the other hand, the slope of storage modulus versus log frequency for a narrow molecular weight distribution should be + 2 ^24^. For pure VM, it is less (1.38 according to Table 1), which is also due to a molecular weight distribution. Understanding the miscibility of polymer blends is crucial for designing materials with specific properties and applications. For miscible blends, the polymer chains of the two components can mix and form a single phase. This results in improved mechanical properties and enhanced compatibility between the polymers.

In contrast, immiscible blends exhibit phase separation, leading to decreased mechanical strength and often a more brittle material. As shown in Fig. 1d, the miscibility of the blend’s parts was examined using the Han plot, which displays the plots of log G′ as a function of log G″. It is generally known that the blends’ Han curves have the same slope as the pure polymer in the low-frequency range. This suggests that there is a case of miscibility between the two components.

Otherwise, they would be immiscible^26^. Figure 1d shows that log G′/log G″ exhibited linear correlation and close slopes for blends of HDPE, 20, 40, and 60% VM. The linear correlation and close slopes suggest compatibility and miscibility of HDPE and VM blends in the molten state for compositions below 60%VM.

On the other hand, a deviation was observed for higher VM contents (80%). This may suggest a solubility limit of about 60% for VM in HDPE, which is in line with ref^22^. A similar phenomenon has been reported for HDPE/ethylene vinyl acetate copolymer^27^.

**Fig. 2:**
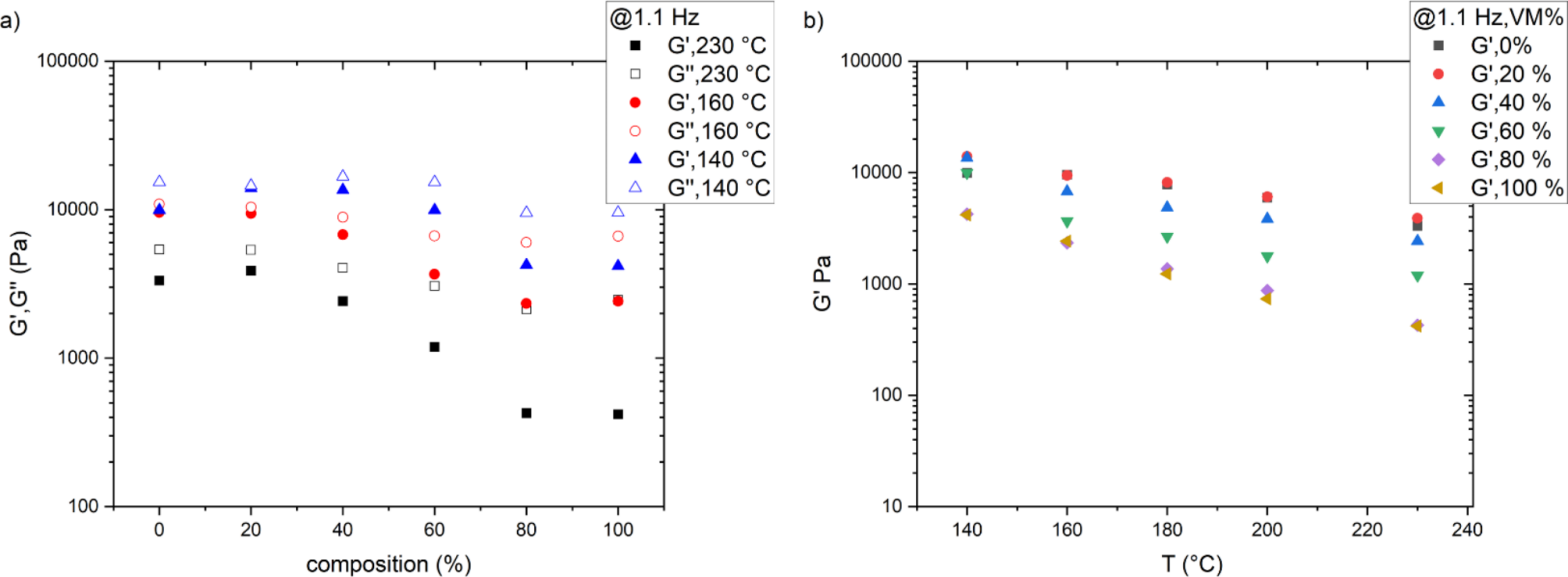
(**a**): Logarithmic scale of storage modulus & viscous modulus at different temperatures in the entire range of blend composition (**b**) Logarithm of the storage modulus G’ at frequency 1 Hz, measured at temperatures falling stepwise from 230 °C to 140 °C, for blends indicated.

Figure 2a presents the variation of G’ and G’’ of HDPE/VM as a function of composition at 1.1 Hz. As the VM content increases, both G′ and G″ decrease, indicating a decrease in the material’s stiffness and viscosity. This trend suggests that adding VM results in a softer and less viscous material, which may affect the material’s mechanical properties and processing characteristics. Moreover, the storage modulus of HDPE and its blends typically decreases with increasing temperature, as demonstrated in Fig. 2b. This is because higher temperatures cause the material to become more flexible and less stiff. As a result, the material can deform more easily under stress, leading to a lower storage modulus. All rheological results indicate that low-content Vistamaxx could be a promising additive for enhancing the performance of HDPE blends in various applications.

### Thermal behavior of HDPE/VM

**Fig. 3:**
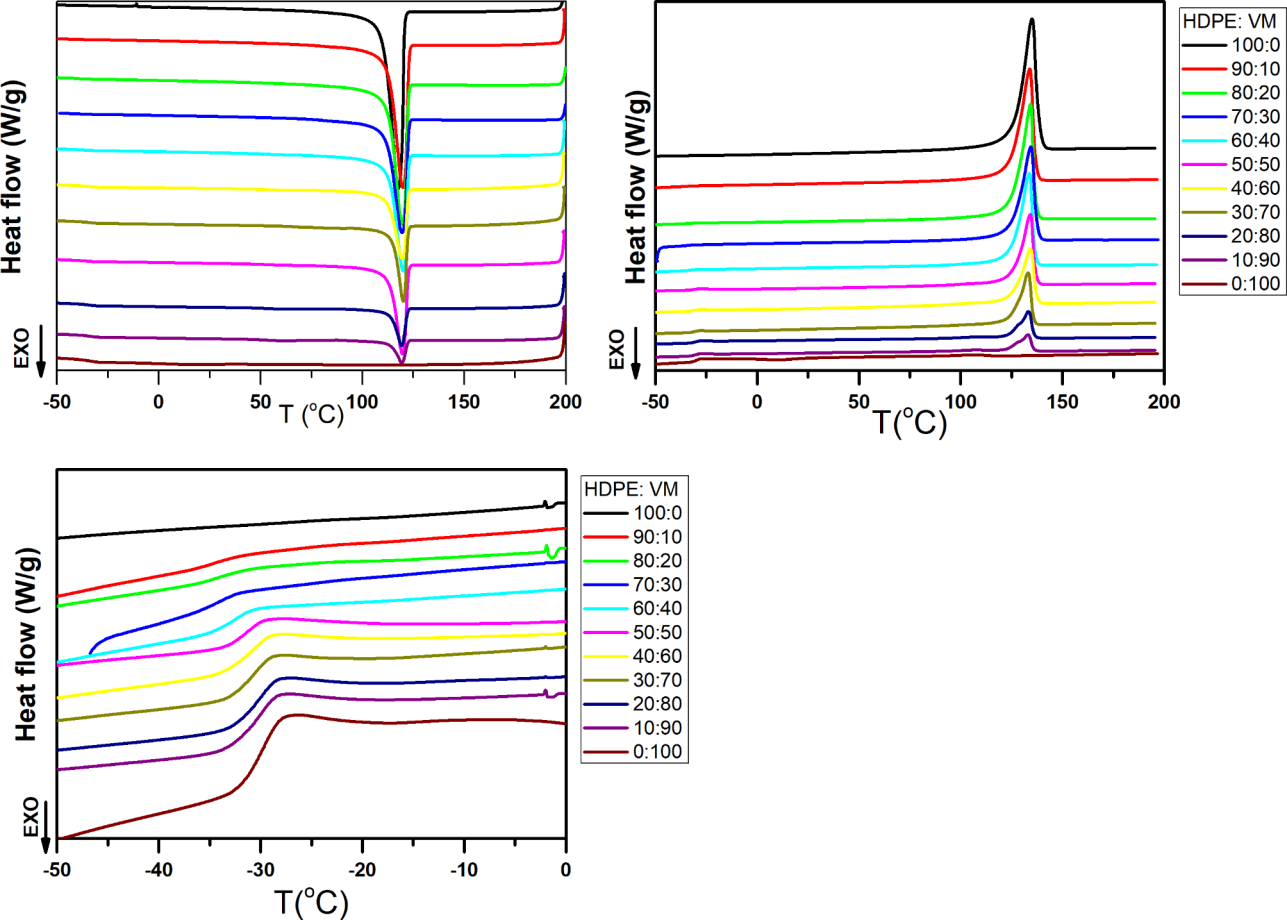
DSC cooling (**a**), second heating (**b**), (**c**) the extended low-temperature scale of second heating curves at 10 °C/min for HDPE/VM blends.

**Fig. 4:**
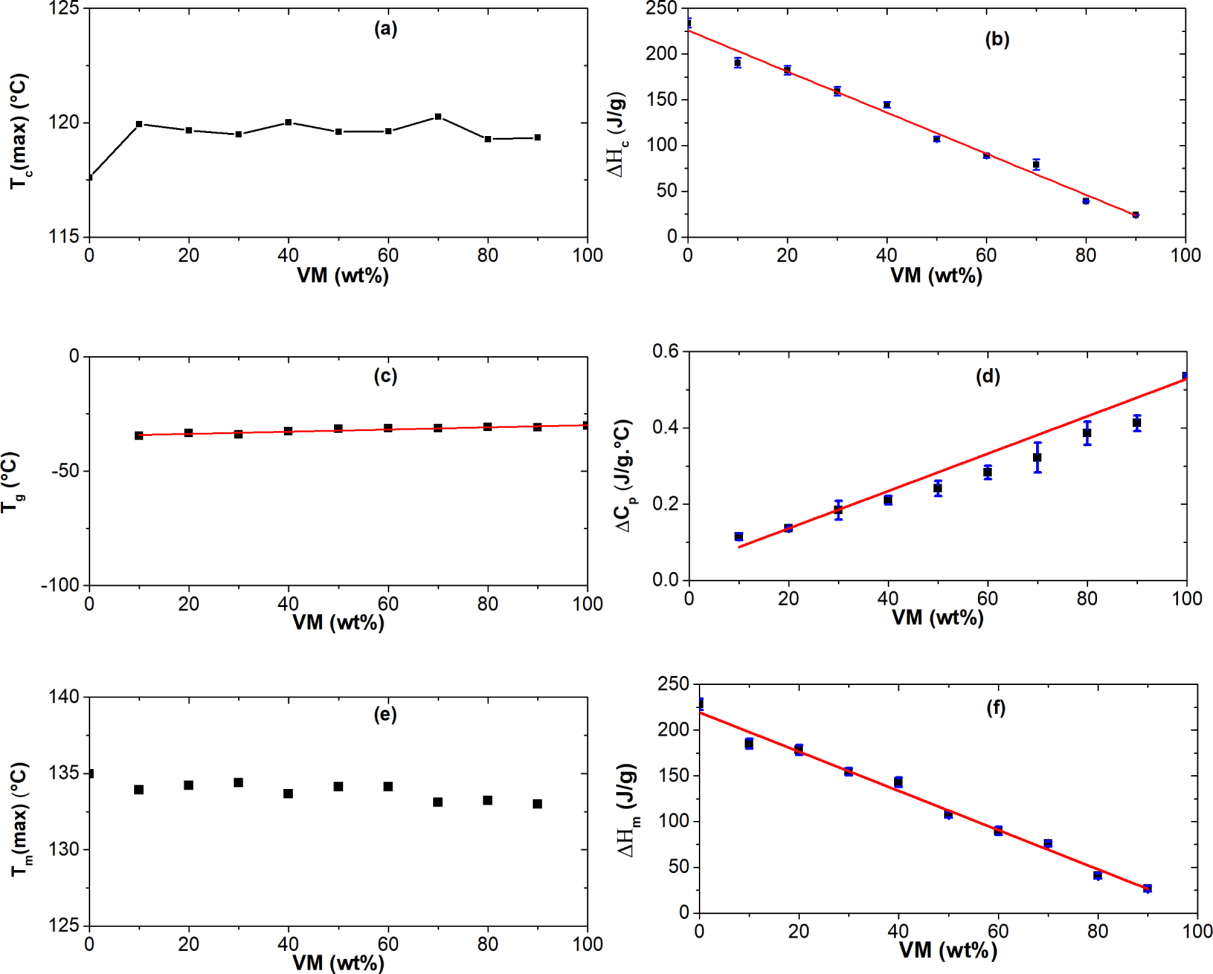
Weight% of VM dependence of DSC transition parameters for HDPE/VM blends; maximum crystallization temperature (**a**), crystallization enthalpy (**b**), glass transition temperature (**c**); change in heat capacity (ΔCP) (**d**); maximum melting temperature (**e**); melt enthalpy (**f**).

The cooling curve for HDPE and its blends with VM is shown in Fig. 3a. Neat HDPE has a prominent exothermic melt crystallization peak at 118 °C, and its crystallization enthalpy is approximately 233 J/g. If 293 J/g is used as the fictive enthalpy of 100% crystalline PE^28^, the crystallinity of HDPE is 80%. On the other hand, a neat VM can be considered an amorphous polymer under this heat treatment since its total crystallinity is around 1%, as discussed in our previous work^21^. Figure 3a shows that for HDPE/VM blends, the HDPE phase’s melt crystallization peak appears at higher temperatures (about 2 °C higher), and this shift is unaffected by the VM content. As VM was molten during HDPE crystallization (its melting temperature was 104 °C^21^), a slight increase in Tc of HDPE in the HDPE/VM blends, as shown in Fig. 4a, may have been caused by the migration of heterogeneities from the VM matrix to HDPE domains. Prudhomme et al. reported that in the absence of interphase interaction, the most straightforward scenario involves total phase separation between the two components and the mostly independent behavior of the two phases concerning crystallization^29^. However, the most frequent finding is that adding a second immiscible polymer, which can function as a nucleating agent, causes an increase in nucleation and growth rates^29^. Greco et al.^30^ also noted that in immiscible blends with EPR, HDPE crystallization temperature shifts by about 3 °C. The literature has also indicated a similar pattern for immiscible blends^31–34^. Furthermore, Fig. 4b shows that the exothermic melt crystallization enthalpy drops systematically.

Figure 3b represents the second heating curve of HDPE and its blend with VM. The scale is extended to a lower temperature to see the glass transition step, as shown in Fig. 3c. The behavior of the neat VM shows that VM exhibits a glass transition at a temperature of − 30 °C, followed by a cold crystallization peak at 11 °C that melts again at T_m_ of 104 °C^21^. The glass transition temperature of neat HDPE was challenging to detect in this temperature range. The glass transition of highly crystalline HDPE is weak; its position is between − 80 and − 120 °C, as mentioned in refs^35–39^. Figure 4c clearly shows a very well-defined glass transition for the blends with a temperature of -30 °C. Interestingly, Tg could be detected in all composition ranges. Weight% independence of glass transition temperature indicates the immiscibility of HDPE and VM after crystallization of HDPE. With increasing the percentage of the VM content, the ∆C_p_ increases linearly, as shown in Fig. 4d. A prominent endothermic melting peak at 135 ^o^C is observed for neat HDPE, as shown in Fig. 2b. Imperfect crystals will be formed due to the presence of VM. The imperfect crystals will melt at slightly lower temperatures (~ 2 °C), as shown in Figs. 3b and 4e. A similar phenomenon was reported in ref^13,40^. DSC analysis of ∆Cp, ∆Hc, ∆Hm composition dependence (Fig. 4b, d and f) shows that the blending of PE and VM is quantitatively reflected in all samples. Accordingly, one can conclude that PE and VM are most probably compatible in the molten state in a composition range of 20 to 60%. However, upon crystallization, different domains of the VM and PE occur distinctively, as discussed above.

### Polarized optical morphology of HDPE/VM

Polarized optical microscopy is a tool to understand HDPE’s compatibility and isothermal crystallization. Shanks et al.^41^ have investigated the compatibility of PEs with PP, concluding that the microphase separation of HDPE and PP occurs during cooling upon crystallization. In this work, the different compositions are subjected to isothermal crystallization from the melt to determine the behavior of HDPE/VM under isothermal crystallization at 119 °C. A temperature of 119 °C allows the HDPE to crystallize in the blend; no crystallization is expected for the VM, as shown in Fig. 3. The VM is 85% propylene units and 15% ethylene units statistically distributed, and only nanocrystals could be formed at low temperatures^42^. (the melting temperature of VM is 104^o^C). For the neat HDPE, small crystallites are detected by other work^43^.

**Fig. 5:**
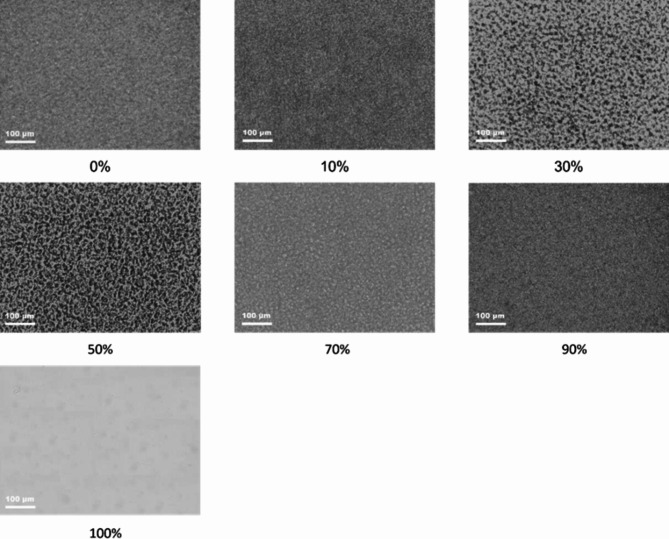
Polarised optical micrographs of HDPE/VM blends at different VM percentages as indicated.

In the composition ranging from 0 to 20% VM, it is seen that fine spherulites of HDPE are found across the sample, as shown in Fig. 5. Figure 5 shows that the PE domains are spread evenly throughout this range, indicating that the mixing and compatibility in the melt are uniform, which aligns with the rheological and DSC results. Figure 5 shows that when the amount of VM was raised by more than 20–50%, coarse spherulites of PE domains could be seen, and the amorphous soft content of VM tended to stick together. At 70–90% VM, the fine spherulites of HDPE are found across the entire composition range.

### Tensile properties of HDPE/VM blends

**Fig. 6:**
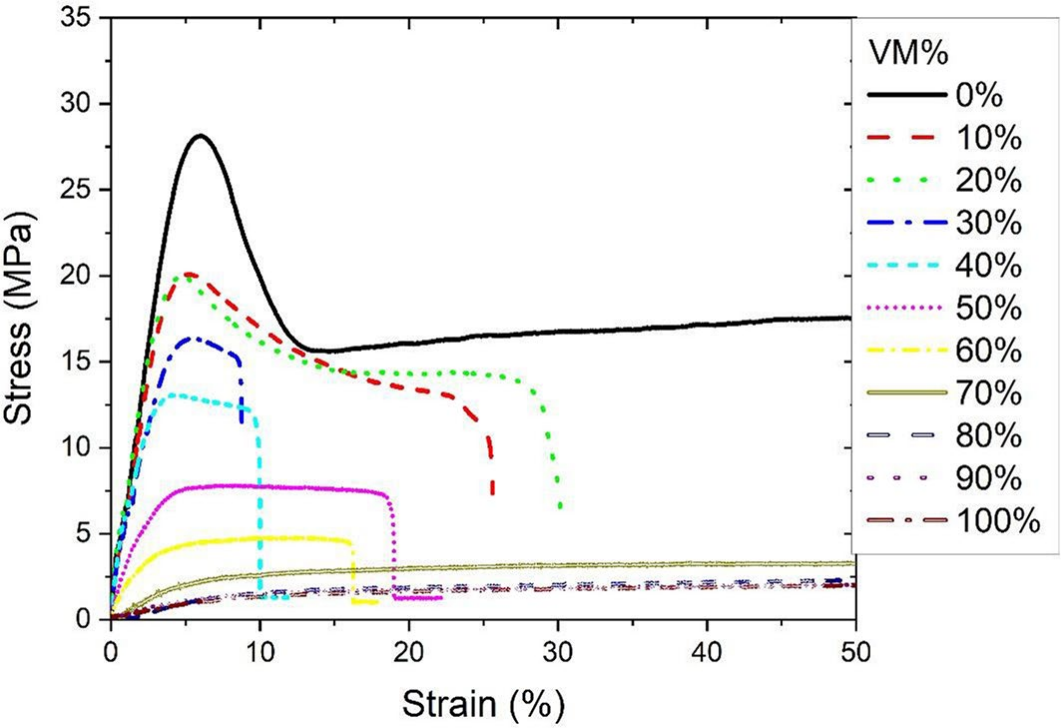
Stress-strain behavior of HDPE/VM blends.

**Fig. 7:**
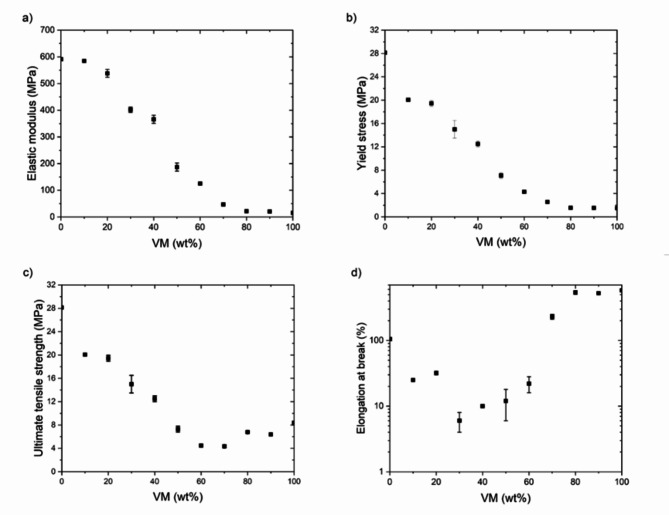
(**a**) elastic modulus; (**b**) yield stress; (**c**) ultimate tensile strength; (**d**) logarithmic scale elongation at break and weight% of VM for HDPE/VM blends.

The stress-strain behavior of HDPE and VM blends is presented in Fig. 6. Increasing the percentage of VM components decreases the elastic modulus and yield stress, as seen in Figs 6 and 7a,b. Adding a 10% VM soft component changes the yield stress value and ultimate tensile strength from 27 MPa to 20 MPa, as represented in Fig. 7b, c. However, the elastic modulus change is linear in increasing the VM; thus, there is no abrupt change, and the system’s compatibility could be proven. However, elongation values at break are considered good enough within the 0–20% composition range (Fig. 6).

This agrees with the polarized optical microscope results, as the PE domains are finely dispersed. A group recently published similar results for HDPE/VM blends and found similar behavior^22^ .They found an increase in the toughness and elongation at break for the HPDE/VM in the composition range of 10% and 20% and then a decrease at the 30% VM content. Liang et al.^13^ discovered that HDPE-rich blends had better mechanical properties than EVA-rich blends. A brittle behavior could be found in the intermediate composition range above VM 20%. Coarse domains of HDPE and microphase separation upon cooling and the coalescence of VM domains lead to different dispersed domains in the solid state. According to Fig. 7, adding VM in this composition range causes the elongation at break to decrease sharply. In the intermediate composition range, blending both PE and VM does not provide good mechanical properties. In our previous manuscript, it was found that PP/VM blends exhibit a critical threshold at a VM concentration of 20%. Below this threshold, the blends demonstrate more brittle behavior. In contrast, above 20% VM, they transition to a more ductile behavior^21^. The difference in the mechanical behavior of PP/VM and HDPE/VM is due to the difference in miscibility in the solid state and melt flow index of both blend’s components. Understanding this behavior helped us discuss the results that were useful in the discussion of ternary blends^20^ and the recycling of polyethylene and polypropylene waste streams.

## Conclusion

The study accomplished successful preparation of blends of polyethylene (HDPE) with Vistamaxx (VM) in the whole composition range, using a single screw extruder on a laboratory scale. A comprehensive investigation was conducted to analyze the rheological characteristics, thermal and crystallization behaviors, and morphological and mechanical properties of HDPE/VM blends. Rheological analysis of HDPE / VM, with VM content higher than 20%, showed lower loss and storage modulus and complex viscosity than pure HDPE. Linear correlation and close slopes of Han Plots (log G′/log G″) for blends of HDPE, 20, 40, and 60% VM suggest good compatibility and miscibility of HDPE and Vistamaxx in the molten state. From DSC experiments, it is found that the crystallization temperature Tc is shifted about 2 °C higher for all blend compositions. This indicates some influence of VM on the nucleation of HDPE.

Regarding the glass transition, the step of the specific heat capacity at Tg appears at − 30 °C for all blend compositions, and its height is strictly proportional to the VM content. For HDPE/VM blends with VM content of 10 and 20%, the elongation at break is about 25%, and stress-strain curves are still similar to those of neat HDPE, whereas, for 30% VM and more, the material seems weak and brittle, and not suitable for technical applications. This finding corresponds to POM images, with a relatively coarse morphology for 30 and 50% VM. From experimental results, it can be inferred that PE and VM are likely to be compatible in molten form within a composition range of 20 to 60%. However, throughout the process of crystallization, discrete domains of the VM and PE occur in a contrasting manner. These results encourage us to explore using VM as a compatibilizer for PP-rich blends of PP/PE.^20^.

## Data Availability

The authors declare that the data supporting the findings of this study are available within the paper. Should raw data files be needed in another format, they are available from the corresponding author upon reasonable request.
